# Improving access to eye care for older people: experiences in South Africa

**Published:** 2016

Older people need greater access to good eye care: 82% of people living with blindness worldwide are over 50, even though they only represent 19% of the world's population.^1^ In South Africa, the 80% of people who are unable to afford expensive private medical insurance are reliant on the state to provide affordable health care.^2^ Older people have access to primary health clinics in the community where many of their health needs are met, free of charge. However, eye care has mainly been provided by private optometrists (usually based in city centres) and at district and university teaching hospitals, which have been struggling to cope with the demand.

Concerned about the lack of eye care provision for older people, the Brien Holden Vision Institute has been working with the provincial departments of health in both KwaZulu-Natal and Gauteng to improve and expand eye care in primary health clinics; this was first achieved in KwaZulu-Natal through the Giving Sight in KZN Project. **Sally Crook** of Seeing is Believing (which has been involved in supporting this work), spoke with **France Nxumalo** about the challenges of replicating the project in Gauteng. France led on the Gauteng project for the institute and has since moved on to a position within the Department of Health in South Africa.

## How did this project come about?

The Brien Holden Vision Institute started collaborating with the Gauteng Department of Health (DOH) in 2010, initially focusing on school eye health. As we started to look at the wider health system, however, one key gap that emerged was access to eye health for older people in Soweto, an area of socio-economic deprivation. There were long waits to see an optometrist and no access to affordable spectacles. Older patients were going directly to the university teaching hospital, resulting in long queues.

The DOH was initially reluctant to adopt the model. However, we conducted active advocacy that culminated in a visit to KwaZulu-Natal by the Gauteng managers. As a result, the DOH agreed to add eye care services to the services already provided by local primary health care centres, where older people were already going for support with all their other health care needs. The DOH agreed to provide space, access to district health information systems, staff (nurses and optometrists) and services.

**Figure:**
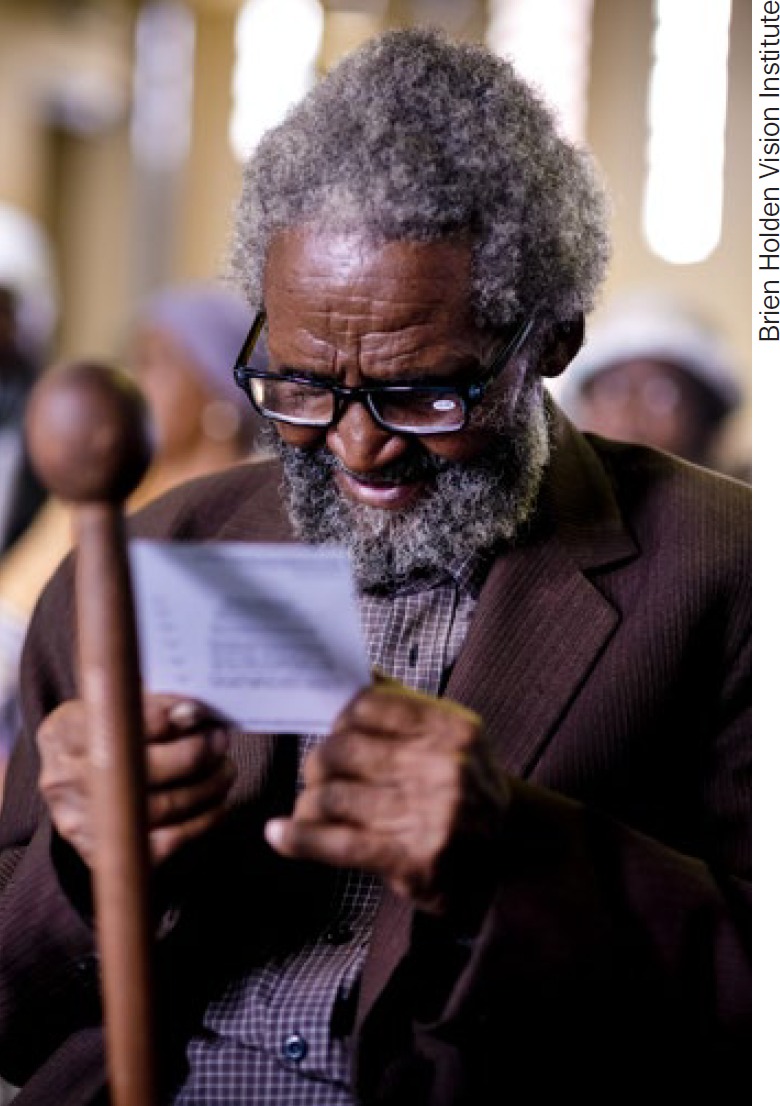
Older people need access to eye health in their own community. SOUTH AFRICA

## How did you support the DoH to understand the eye care needs of older people?

Advocacy was key. We shared prevalence data demonstrating the eye care needs of older people with senior managers. We also organised a visit to KwaZulu-Natal to show managers the primary eye care model that was already working there. The Gauteng managers were then able to discuss the model with their KwaZulu-Natal counterparts and saw how the programme was implemented at both primary and district levels.

## What happened once the Gauteng DOH agreed to the project?

The nurses working in the clinics had to be trained in primary eye care so they would be able to screen and refer the older patients. Optometrists had to be recruited and access to equipment and consumables also had to be addressed. There are already full-time optometrists providing services at 13 Soweto primary health care centres, with plans to have a full-time optometrist serving each of the remaining primary health centres in the province. At some facilities, where the nurses are particularly busy, health promoters (who were already employed in the primary health care centres) were trained to assist with screening.

## How has the new model improved access for older people?

Older people are already visiting primary health care centres for their other health care needs, so they are now able to access refractive error and screening services very easily. There are clear referral links with other chronic clinics, such as diabetes and hypertension clinics, and access to cataract services has improved thanks to referral to the local district hospital. Only patients with more complicated needs are referred, within the health system, to the provincial hospital. Reading spectacles are dispensed free of charge although prescription spectacles must be paid for. The government is currently working on a sustainable model of spectacle supply.

## Are people using the new services?

The number of older people seeking care at the primary health care centres has increased and is still increasing. People like the new eye care services and use them as access is much easier – the services are nearer to their homes and wait times are shorter. This improvement in access at the primary and district level has also eased pressure at the university teaching hospital level, which has been noted by the DOH.

## Is this model sustainable?

Yes, it is sustainable because it is integrated into the DOH system, which means that the staff and facilities are all paid for by the government. Spectacle supply remains an issue as government staff at primary level facilities cannot accept cash for spectacles or services.

## What are the key things you have learned in this process?

- Advocacy is important and needs to be continued. We had to provide evidence to back up the model proposed. Showing senior managers what worked helped to engage them in the issue.
- The government needs to be involved early on. Their commitment was needed to employ a new cadre of staff (optometrists) and this also prevents future collapse of the project.
- Be adaptable and be prepared to change strategy. For example, when the primary health centre nurses did not have enough time during their working day to deliver all the necessary eye care, we trained the health promoters to take on some of the eye care workload.
